# Educating Youths to Make Safer Choices: Results of a Program Evaluation Study

**DOI:** 10.5539/gjhs.v4n2p77

**Published:** 2012-03-01

**Authors:** Donna M. Wilson, Carrie Chamberland, Jessica A. Hewitt, Donna Wilson

**Affiliations:** Faculty of Nursing, ECHA Building, University of Alberta Edmonton AB T6G 1C9, Canada Tel: 780-492-5574 E-mail: donna.wilson@ualberta.ca; Misericordia Community Hospital, Edmonton AB T5R 4H5, Canada Tel: 780-735-2890 E-mail: Carrie.Chamberland@albertahealthservices.ca; Faculty of Nursing, Third Floor ECHA, University of Alberta Edmonton AB T6G 1C9, Canada Tel: 780-492-5574 E-mail: jahewitt@ualberta.ca

**Keywords:** Injury prevention, Adolescents, Nursing roles, Program evaluation, Research

## Abstract

Injuries are a leading cause of childhood death and disability. Many injuries are a result of youths taking risks and not avoiding risky situations. An educational program to reduce adolescent injury risk (Prevent Alcohol and Risk-Related Trauma in Youth) has operated out of the Misericordia Hospital in Edmonton Canada since 1992. This reality-based program was evaluated to see if it was impacting program participants. An increase in correct answers for some knowledge, behavior, and attitude questions were found at one week and one month following this 1-day reality-based program. This program was thus considered as having some relevancy in educating grade-9 youths. Although a longitudinal study is needed to determine if this relevancy is long term, this study highlights the importance of reality-based public health programs.

Injuries are a leading cause of childhood death and disability in many countries today (Ekeh *et al.*, 2011; Lammers *et al.*, 2011; National Center for Chronic Disease Prevention and Health Promotion, 2010; Public Health Agency of Canada, no date b). Adolescents aged 15-19 have the highest risk of injury-related deaths, with these often a result of motor vehicle collisions (Ekman *et al.*, 2005; Safe Kids Canada, 2006). Survivors often face permanent disabilities, both physical and emotional. With injuries also a common reason for youth hospitalizations (Ekman *et al.*, 2005; Public Health Agency of Canada, no date a), the impact of injuries on young people, their families and friends, and the healthcare system as a whole should be considered as not only very serious but highly regrettable. Many injuries and deaths among adolescents are a result of their taking risks and their not avoiding risky situations (Alberta Centre for Injury Control and Research, 2010; Canadian Centre on Substance Abuse, 2011; Ekeh *et al.*, 2011; Ekman *et al.*, 2005; Lammers *et al.*, 2011).

A growing number of educational programs are being developed to address this major public health issue (Currie *et al.*, 2004; Elliott, Orr, Watson, & Jackson, 2005; Klassen, MacKay, Moher, Walker, & Jones, 2000; Nilsen & Yorkston, 2007; Peleg Neumann, Friger, Peleg, & Sperber, 2001; Shope, Elliott, Raghunathan, & Waller, 2001). One such program, the Prevent Alcohol and Risk-Related Trauma in Youth (P.A.R.T.Y.) program, was initiated at the Sunnybrook Hospital in Toronto Ontario Canada in 1986. There are over 60 P.A.R.T.Y. programs across Canada now; with programs also having been initiated in the United States, Japan, and Australia. The aim of this program is to increase youth knowledge about risks, and to change the attitudes and behaviors of adolescents so they are less likely to suffer risk-related trauma of all kinds.

One P.A.R.T.Y. program has operated since 1992 out of the Misericordia Hospital, a 350 bed full service acute care hospital, in Edmonton Alberta Canada. A Registered Nurse, who has worked in hospital emergency departments for many years, is the program coordinator. Although positive comments over the years have been received from youth participants, presenters, parents, and teachers; the program has never been formally evaluated. This program needed to be evaluated to ensure it was impacting student participants as expected. Specifically, a research study was designed to answer one question: What are the knowledge, attitudes, and behaviors about risk-related trauma among grade-9 students; and are these impacted by the P.A.R.T.Y. program?

## 1. Background

Since its inception in 1992, over 70,000 mainly grade-9 students from around Alberta have participated in the Misericordia Hospital program. One of the chief features of this one-day (5.5 hour) program is the direct involvement of nurses, paramedics and other emergency medical services personnel, police officers, community volunteers, and trauma victims. Slide and video presentations, role-playing, and discussions are featured. The discussions are led by injury victims, emergency room nurses, police officers, and paramedics. All talk frankly about their roles, experiences, and frustrations over needless accidents. Issues arising from alcohol and drug consumption, and peer pressure are highlighted throughout the day.

Injury survivors also speak about what it is like to live with brain, spinal cord, and other permanent disabling injuries. The program is designed so students can engage in candid discussions with injury survivors and see firsthand some of the daily challenges of living with brain, spinal cord, and other serious injuries. Students have the opportunity to use a wheelchair and eat lunch as if they were a quadriplegic. Through this reality-based education, youths are encouraged to consider the possible consequences before engaging in risky actions and not avoiding risky situations. This program also emphasizes personal choice, as children at this age are becoming increasingly independent from their parents and teachers (Newman, 1989). They typically prize independence and the right to makes choices about their own lives (Newman, 1989). Unfortunately, they are also highly susceptible to peer pressure (Coronges, Stacy & Valente, 2011). Alcohol and drug use is common among youths (Canadian Centre on Substance Abuse, 2011; Reavley *et al.*, 2011). Research on substance abuse indicates that alcohol and drug use that is initiated as youths can continue into later life (Oesterle, Hawkins & Hill, 2011). Consequently, prevention programs that are designed specifically for younger adolescents are of particular relevance (Oesterle, Hawkins & Hill, 2011).

Some evaluations of programs designed to reduce substance abuse and accidents among youths have been conducted, with these published articles available to influence the design of a program evaluation research study. The findings from these program evaluations are also helpful for being optimistic about educational programs. A systematic review of educational programs aimed at youth alcohol reduction, conducted by Foxcroft *et al*. (2011), also raises optimism about educational programs. In this review, 36 of the 56 studies revealed the program was effective.

More specifically, Robertson *et al*.’s (2011) 18-session prison education program aimed at reducing unprotected sex when alcohol or drugs were used was revealed as effective when participants were tested nine months after discharge. A study of young women who were assigned to a control or brief intervention program that was similarly aimed at reducing unprotected sex also revealed considerable value from this educational program (Essien *et al.*, 2011). Those in the intervention group were five times more likely to suggest that their new male partners use condom. The program was video-based, and so considered simple and easy to conduct (Essien *et al.*, 2011). Driving has also been the focus of research, with a Drive Alive program for teens evaluated by Ekeh *et al*. (2011). This program is four weeks in length and involves a total of 10 hours of mock trauma sessions, drug and alcohol education, and contact with former trauma patients and their families, and state troopers. The program was credited with reducing the incidence of driving-related convictions over the first six months after program completion. Lammers *et al*.’s (2011) evaluation of a program to prevent binge drinking among young adolescents is also notable. This study, involving 13-15 year old at-risk students who attended two 90-minute educational sessions, indicated that few would continue to binge drink in the future (Lammers *et al.*, 2011). Vincus *et al*.’s (2011) recent study of a short educational program designed for 5th grade students did not, however, reveal any intervention effects related to reduced substance use. This program was school based, as the curriculum was designed to include substance abuse and risk information. In contrast, Cooper and Al-Alami’s (2010) study involving an external group providing school education in the form of talks on smoking, drug and alcohol abuse, healthy eating and STDs revealed program effectiveness. The researchers indicated that much of the effectiveness of this program was due to an outside group providing the education.

## 2. Research Methods

After research ethics approval and hospital administrative approval was gained for this study, and a research grant was obtained to gain research assistance and to cover photocopying and mail costs, a multiple-choice questionnaire was developed by the Program Coordinator. This questionnaire was pilot tested with select nursing staff and youths, with improvements made for readability and content issues (questionnaire appended). After this development, the contact person at all schools who were planning to have their grade-9 students attend the program in the fall of 2009 were contacted and told about the study. Specifically, these contacts were told that the hospital wanted to evaluate the program and that this voluntary study involved the same questionnaire administered three times; the first on the morning immediately prior to the program starting, the second one week after the program, and the third one month after the program. The research ethics committee would not allow students to be named or numbered or tracked, and with voluntary participation, this requirement meant that the research team could not track an individual’s scores from the first through third questionnaire, nor determine if the same individual completed all three questionnaires or not. Group data instead were thus compared from the first to second questionnaire to determine for improved knowledge, attitudes, and reported behaviors following the program. Group data were also compared from the first to third questionnaire to determine if these improvements were retained over a one month period.

Three school representatives agreed to ensure that all of their attending students and their parents received an information letter and consent form. This form needed to be signed by one or both parents before their child could take part in this study, and research participation was not mandatory for the students taking the program. As expected some students (numbers outlined later) from the three schools completed it immediately prior to the program, one week later, and one month after the program. As indicated, a pre-post analysis of data from all 385 questionnaires was done to see if knowledge, attitudes, and reported behaviors about risk-related trauma increased and remained improved for one month. The SPSS (version 17) program was used to analyze data, with the consent forms locked away and only accessible to the research team. The questionnaire did not ask for the student’s name, and so this data was not entered onto the computer spreadsheet. Simple descriptive statistics, chi-square analyses, and Spearman correlations were used to summarize the data and assess for differences in responses.

## 3. Results

A total of 385 surveys were returned; 140 pre-program surveys, 123 1-week surveys, and 122 1-month surveys. The return rate for the first survey was 64%, with an attrition of 12-13% after. Because no identifying information about the students could be collected, it is not known whether students completed more than one survey.

Table 1 shows the percent who selected the correct answers to eight (multiple choice) knowledge questions. An increase in correct answers for three questions was noted from the pre-program to 1-week survey, and with this increase retained at one month. The remaining questions had high scores in all three surveys, with no improvements identified.

**Table:** Table 1. Percentage of correct answers to knowledge questions in the three surveys

Question (correct answer)	First Survey N (%)	Second Survey N (%)	Third Survey N (%)
A1 (E) *	110 (78.6)	105 (85.4)	107 (87.7)
A2 (E) *	89 (63.6)	85 (69.1)	87 (71.3)
A3 (A)	133 (95)	111 (91)	106 (88.3)
A4 (C) *	44 (31.7)	50 (41.3)	46 (37.7)
A5 (A)	102 (72.9)	83 (67.5)	86 (70.5)
A6 (B)	107 (77)	102 (83.6)	94 (77)
A7 (E)	127 (90.7)	113 (92.6)	110 (90.2)
A8 (D)	66 (47.5)	65 (54.2)	61 (50.4)

* denotes a statistically-significant increase in knowledge

Six questions assessed risk-related behaviors. As shown in Table 2, the answers to two of these questions revealed improved behaviors which were retained to the 1-month point. Four questions did not reveal sustained improvements, but missing data were common for these questions.

**Table:** Table 2. Changes in knowledge, behaviors, and attitudes over duration of study

Knowledge Questions	Statistical Test Result
A1 * (improved)	χ^2^=17.918, df=8, p=.022
A2 * (improved)	χ^2^=15.746, df=8, p=.046
A3	χ^2^=7.573, df=6, p=.271
A4 * (improved)	χ^2^=18.891, df=8, p=.015
A5	χ^2^=2.527, df=8, p=.960
A6	χ^2^=3.337, df=8, p=.911
A7	χ^2^=5.038, df=8, p=.754
A8	χ^2^=3.679, df=8, p=.885
**Behavior Questions**	
B1	r^2^=-.048, p=.359
B2	r^2^=.074, p=.175
B3	r^2^=.001, p=.990
B4* (improved)	r^2^=.135, p=.019
B5* (improved)	r^2^=.120, p=.037
B6	r^2^=-.007, p=.898
**Attitude Questions**	
Ca	χ^2^=10.178, df=8, p=.253
Cb	χ^2^=4.456, df=8, p=.814
Cc	χ^2^=7.871, df=8, p=.446
Cd	χ^2^=14.622, df=8, p=.067
Ce * (improved)	χ^2^=15.505, df=8, p=.050
Cf * (improved)	χ^2^=24.527, df=8, p=.002
Cg * (improved)	χ^2^=17.232, df=8, p=.028
Ch	χ^2^=4.230, df=8, p=.836

* denotes statistically-significant changes across the three surveys

**Table 3:** Percentage of unanswered behavior questions by survey

	Percentage (%) of Unanswered Questions		
Questions With no Answers	Pre-Program	One-week Post	One-month Post

B1	2.9	0.8	5.7
B2	12.9	8.9	11.5
B3	30.7	19.5	22.1
B4	22.9	17.1	23.8
B5	25.7	15.4	22.1
B6	22.9	12.2	15.6

Eight questions assessed risk-related attitudes. As shown in Table 2, improved attitudes were found through three questions, with these improvements retained at the 1-month point. Some improvements were found for the remaining five questions, but these were either insignificant changes or not retained over one month.

## 4. Discussion

This study revealed a number of improvements in knowledge, attitudes, and reported behaviors; a major reason for continuing this program and possibly expanding it to other schools offering grade-9 education or education to youths somewhat younger or older. The hospital setting, timing (in grade-9, when most students are aged 13-14 and so before they can drive independently), content, and chosen teaching/learning methods could have all contributed to these improvements. Among these factors, the hospital may have been key for providing a realistic setting or environment for learning. Few students at this age and their siblings or parents are likely to have been hospitalized in the past, as less than 10% of Canadians are hospitalized each year now (Canadian Institute for Health Information, 2009). Having an experienced Program Coordinator who is an emergency room nurse, and who has run this program for many years, could have also been another major factor for ensuring an effective program. At this age, gaining and holding student attention is highly relevant to their learning. In addition, the police, paramedics, injured persons, and other volunteers (including the teachers who attended) were all potentially highly important to learning, as they contributed to frank and open discussions such that the risks of substance abuse were made clear to students. Regardless of which one or more factors contributed to the noted improvements in knowledge, attitudes, and/or reported or intended behaviors, this reality-based risk-reduction program was identified through this survey as having value and has having met the overall objective - to potentially reduce injuries and deaths through improved knowledge, attitudes, and intended behaviors.

It was notable that the risk-related knowledge of students increased and was sustained over one month. Many of the students, however, had relatively high initial knowledge. This high initial knowledge indicates students pay attention to risk information from their parents, peers, schools, communities, or other sources. Improvements in some reported risk-related behaviors were also found, with these improvements suggesting students assumed more responsibility for being safe. The students were not presented with an exhaustive list of behaviors to report on; so it is hopeful that if one improvement was made, such as seatbelt buckling up, other risk-reduction behavior improvements could also be occurring.

The low response rate and answers to some behavior questions is of concern, however. It is possible that some questions were not relevant to all or many of these students, as not all would be with older persons who could be potentially drinking and driving. It is also possible that students are not comfortable revealing the truth about their behaviors. Regardless, the responses of students to attitude questions showed a number of significant immediate and sustained improvements. These improvements are of great interest; as attitudes impact behaviors, and attitudes can be highly resistant to change. Caution must be taken, however, in putting too much confidence in the findings of this study and generalizing the findings to other students of this age or to other risk-reduction programs. The Hawthorne effect, a temporary change in behavior in response to research conditions, could have impacted student responses. In addition, long-term follow-up studies are needed to see if life-long attitudes, knowledge, and behaviors that reduce risk are gained through targeted programs such as the P.A.R.T.Y program.

In conclusion, the findings of this study indicate a reality- and hospital-based P.A.R.T.Y. program has positive impacts on the risk-related knowledge, attitudes, and behaviors of grade-9 students. These are most welcome findings as these students will shortly enter the prime age (15-19) for injuries and deaths due to alcohol and substance abuse. This study suggests the importance of targeted community-based programs to equip youths with the information and attitudinal or behavior tools to minimize or prevent harm to themselves and others. A carefully designed, concentrated reality-based program that is held in a hospital may be key, however, for impacting youth risk-related knowledge, behaviors, and attitudes.

## Appendix

Program Evaluation Survey Date completed:

For each of the following questions, please indicate the most correct answer by circling the letter in front of your choice.

### A. Knowledge

#### A. 1. When you suffer a permanent disability as a result of an injury, would you probably have…

a) to change major aspects of your lifestyle
b) effects on your relationships with your family and friends
c) to reestablish yourself in your community after hospitalization
d) to make changes to your physical environment
e) all of the above

#### A. 2. To “Buckle Up” means to…

a) wear a seatbelt while riding or driving a motor vehicle
b) wear a bike helmet with a strap fastened while biking
c) wear a personal flotation device when in a boat
d) fasten the climbing harness when rock climbing
e) all of the above

#### A. 3. To “drive sober” means to…

a) not drink alcohol or do drugs before driving a car or snowmobile
b) not drink alcohol or do drugs before operating a boat
c) not be over tired while driving
d) not be distracted by friends while driving

#### A. 4. Most injuries to Canadian youth …

a) only occur to those who engage in “extreme sports”
b) are “accidents” and cannot be prevented
c) are predictable and preventable
d) are totally avoidable if you behave safely
e) none of the above

#### A. 5. To “look first” means to…

a) make sure everything is safe before proceeding
b) examine any situation that you are about to enter for the potential risks
c) check the depth of the water before diving in
d) make sure the ice is frozen solid before walking or driving on it
e) none of the above

#### A. 6. When you experience a spinal cord or head injury…

a) you will either die or completely recover
b) your lifestyle may be permanently changed
c) you will only be affecting your own life
d) you will probably require a short rehabilitation period
e) none of the above

#### A. 7. To “wear the gear” means to…

a) wear any eye shield on your hockey helmet
b) wear a helmet when snowboarding
c) wear a bike helmet
d) wear wrist guards or knee pads when in-line skating
e) all of the above

#### A.8. To “get trained” means to…

a) copy what the pros do
b) take driver training before taking your driving test
c) learn from your friends or parents before trying a new activity
d) receive professional instruction before trying a new sport, job or activity
e) all of the above

### B. Your Past Behavior:

In the past three (3) months, please indicate how often you have performed each of the following behaviors by using the rating scale following each item. If you did not perform the behavior during the last three (3) months (eg: did not drive a vehicle, did not go to a party), then circle “N/A” which stands for “not applicable.”

B. 1. I wore my seatbelt while riding as a passenger in a motor vehicle:

1. 2. 3. 4. 5. 6.

Never Sometimes About half the time Most of the time All the time N/A

B. 2. I rode in a vehicle (eg. car, motorcycle, snowmobile, boat) that was being driven by someone under the influence of alcohol or drugs.

1. 2. 3. 4. 5. 6.

Never Sometimes About half the time Most of the time All the time N/A

B. 3. Before going to a party, I planned how I would get home to avoid riding with an impaired driver.

1. 2. 3. 4. 5. 6.

Never Sometimes About half the time Most of the time All the time N/A

B. 4. I operated a piece of equipment (eg: car, motorcycle, snowmobile, tractor or farm equipment, chainsaw, power tool), while under the influence of alcohol or drugs.

1. 2. 3. 4. 5. 6.

Never Sometimes About half the time Most of the time All the time N/A

B. 5. I looked out for the welfare of a friend of family member who was in a risky situation.

1. 2. 3. 4. 5. 6.

Never Sometimes About half the time Most of the time All the time N/A

B. 6. I told my friends or family members when I saw they were facing a serious risk in a situation.

1. 2. 3. 4. 5. 6.

Never Sometimes About half the time Most of the time All the time N/A

### C. Your Attitudes

Please indicate your thoughts about each of the following questions by using the rating scale following each item. Circle one answer.

C. 1. It is my life and if I take risks, and if I choose to take risks, it is my business because I am only endangering myself.

1. 2. 3. 4. 5.

Strongly disagree Disagree Neutral Agree Strongly agree

C. 2. Life is about identifying the risks that I face in everyday life and choosing how to manage them.

1. 2. 3. 4. 5.

Strongly disagree Disagree Neutral Agree Strongly agree

C. 3. Most teens do not encounter risky situations in their daily lives.

1. 2. 3. 4. 5.

Strongly disagree Disagree Neutral Agree Strongly agree

C. 4. If I am injured while riding as a passenger with a driver who is impaired, it is my responsibility because I chose to take a ride.

1. 2. 3. 4. 5.

Strongly disagree Disagree Neutral Agree Strongly agree

C. 5. My actions can result in a permanent consequence for me.

1. 2. 3. 4. 5.

Strongly disagree Disagree Neutral Agree Strongly agree

C. 6. My actions can result in permanent consequences for others (my parents/guardians, family or friends).

1. 2. 3. 4. 5.

Strongly disagree Disagree Neutral Agree Strongly agree

C. 7. Injuries are a problem for some teens, but I don’t believe that I am personally at risk.

1. 2. 3. 4. 5.

Strongly disagree Disagree Neutral Agree Strongly agree

C. 8. I can make choices about many of the risks that might lead to me being injured or killed.

1. 2. 3. 4. 5.

Strongly disagree Disagree Neutral Agree Strongly agree

**Thank You for Participating In This Survey**
